# Human NK Cells and Herpesviruses: Mechanisms of Recognition, Response and Adaptation

**DOI:** 10.3389/fmicb.2019.02297

**Published:** 2019-10-04

**Authors:** Mariella Della Chiesa, Andrea De Maria, Letizia Muccio, Federica Bozzano, Simona Sivori, Lorenzo Moretta

**Affiliations:** ^1^Department of Experimental Medicine (DIMES), School of Medical and Pharmaceutical Sciences, University of Genoa, Genoa, Italy; ^2^Centre of Excellence for Biomedical Research, University of Genoa, Genoa, Italy; ^3^Department of Health Sciences (DISSAL), School of Medical and Pharmaceutical Sciences University of Genoa, Genoa, Italy; ^4^Clinica Malattie Infettive, Ospedale Policlinico San Martino IRCCS, Genoa, Italy; ^5^Laboratory of Tumor Immunology, Department of Immunology, IRCCS Ospedale Bambino Gesù, Rome, Italy

**Keywords:** NK cells, Herpesvirus, activating receptors, TLRs, memory responses, viral reactivation

## Abstract

NK cells contribute to early defenses against viruses through their inborn abilities that include sensing of PAMPs and inflammatory signals such as cytokines or chemokines, recognition, and killing of infected cells through activating surface receptors engagement. Moreover, they support adaptive responses via Ab-dependent mechanisms, triggered by CD16, and DC editing. Their fundamental role in anti-viral responses has been unveiled in patients with NK cell deficiencies suffering from severe Herpesvirus infections. Notably, these infections, often occurring as primary infections early in life, can be efficiently cleared by NK, T, and B cells in healthy hosts. Herpesviruses however, generate a complicated balance with the host immune system through their latency cycle moving between immune control and viral reactivation. This lifelong challenge has contributed to the development of numerous evasion mechanisms by Herpesviruses, many of which devoted to elude NK cell surveillance from viral reactivations rather than primary infections. This delicate equilibrium can be altered in proportions of healthy individuals promoting virus reactivation and, more often, in immunocompromised subjects. However, the constant stimulus provided by virus-host interplay has also favored NK-cell adaptation to Herpesviruses. During anti-HCMV responses, NK cells can reshape their receptor repertoire and function, through epigenetic remodeling, and acquire adaptive traits such as longevity and clonal expansion abilities. The major mechanisms of recognition and effector responses employed by NK cells against Herpesviruses, related to their genomic organization will be addressed, including those allowing NK cells to generate memory-like responses. In addition, the mechanisms underlying virus reactivation or control will be discussed.

## Introduction

Human NK cells are innate lymphocytes that rapidly provide defenses against tumors and viral infections allowing pathogen elimination or limiting viral spread (Vivier et al., 2011; Della Chiesa et al., 2014b). Their fast responses mainly rely on the expression of multiple germ-line encoded activating receptors among which natural cytotoxicity receptors (NCRs) and NKG2D play the most relevant role in the recognition and killing of infected cells (Bottino et al., 2000; Moretta and Moretta, 2004; Lanier, 2015). The responses elicited by activating receptors are integrated and balanced by the engagement of inhibitory receptors mainly depending on those specific for HLA class I (HLA-I) molecules that include the Killer Ig-like Receptors (KIRs), able to distinguish among allotypic determinants of HLA-A, -B and -C (Bottino et al., 1996; Parham, 2005), the CD94/NKG2A heterodimer, specific for the non-classic HLA-I molecule HLA-E (Braud et al., 1998), and LILRB1 (or CD85j/ILT-2) broadly recognizing HLA-I alleles (Colonna et al., 1997).

Upon infection many viruses, including Herpesviruses, target T cell function via specific interactions with TCR and HLA-I molecules. Indeed, several viral products interfere with host TAP proteins and HLA-I expression, leading to reduced CTL-mediated recognition of infected cells, and decreased naïve T cell activation (Hill et al., 1995; Imai et al., 2013; Schuren et al., 2016). Conversely, downregulated HLA-I expression renders infected cells susceptible to NK-cell killing (Huard and Fruh, 2000; Tortorella et al., 2000). However, activating counterparts of HLA-I-specific receptors, namely activating KIRs (aKIRs), and CD94/NKG2C can also importantly contribute to defense against virus (Della Chiesa et al., 2015).

Human NK cells are usually divided in two major populations, the CD56^bright^ subset expressing NKG2A, lacking KIRs and CD16 (i.e., a low affinity Fcγ Receptor) and the CD56^dim^ subset expressing high CD16 and variable proportions of KIRs, NKG2A, LILRB1, CD57, and NKG2C (Cooper et al., 2001; Caligiuri, 2008; Freud et al., 2017). These two subsets differ in their proliferative potential, cytotoxic activity, cytokine production, and homing to peripheral tissues (Moretta, 2010; Castriconi et al., 2018) thus offering different anti-viral defenses. Notably, CD56^dim^ NK cells, besides high cytotoxicity, can also rapidly produce IFN-γ and TNF-α upon receptor-induced cell triggering (De Maria et al., 2011).

The critical role of NK cells in viral defense has been disclosed by the higher susceptibility to viral infections, caused primarily by Herpesviruses, in individuals affected by congenital immunodeficiencies in which NK cells are absent or defective (Orange, 2002; Etzioni et al., 2005; Notarangelo and Mazzolari, 2006; Mace and Orange, 2019). Herpesviruses are a family of dsDNA viruses, divided in three subfamilies, i.e., α- (HSV-1, HSV-2, and VZV), β- (CMV, HHV6, and HHV7) and γ-Herpesvirus (EBV and KSHV), that differ for their genetic content, infection sites and pathogenesis, while sharing the ability to persist in the host in a latency status after resolution of a primary infection (De Pelsmaeker et al., 2018). The mechanisms by which Herpesviruses establish and maintain latency have not been completely elucidated.

In an evolutionary perspective, our immune system and Herpesviruses have co-evolved influencing reciprocally. During this process the generation of several viral immunoevasion mechanisms has been favored. Most of these mechanisms aim at limiting and suppressing NK-cell responses, which point again to the relevance of these lymphocytes in Herpesvirus control. Although viral immunoevasion strategies are crucial in NK-Herpesvirus interactions, they will not be specifically addressed here and have been exhaustively reviewed elsewhere (Corrales-Aguilar et al., 2014; De Pelsmaeker et al., 2018).

On the other hand, the host-Herpesvirus interaction has exerted a strong pressure on our immune system likely favoring the generation of unexpected memory responses by NK cells and their adaptation to Herpesviruses, in particular to CMV (Muntasell et al., 2013; Sun et al., 2014).

## Overview of the Main Activating Receptors Regulating Nk-Mediated Recognition and Effector Responses to Herpesvirus

**FIGURE 1:**
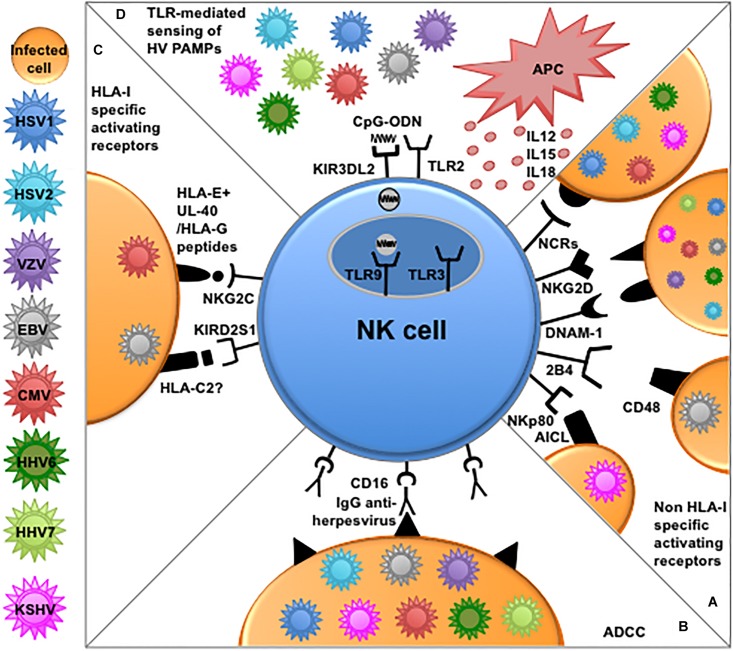
NK cell-mediated mechanisms of recognition and responses to Herpesviruses. **(A)** Several non-HLA-I-specific activating receptors and co-receptors, i.e., NCRs, NKG2D, DNAM-1, 2B4, and NKp80 play an important role in the elimination of cells infected by different Herpesviruses through the recognition of cellular ligands expressed on target cells. **(B)** NK cells can efficiently kill opsonized Herpesvirus-infected cells through antibody-dependent cellular cytotoxicity (ADCC) via CD16 engagement by the Fc fragment of anti-viral immunoglobulins. **(C)** NKG2C and aKIRs play a role mainly in the recognition of CMV-infected cells. The underlying recognition mechanisms are based on interactions with cognate HLA-I molecules. NKG2C shows enhanced interaction to HLA-E presenting peptides derived from viral UL-40 or HLA-G leader sequences, while, among aKIRs, KIR2DS1 seems to better recognize HLA-C2, modified upon CMV infection or presenting EBV-derived peptides. **(D)** NK cells express different functional TLRs involved in the recognition of PAMPs derived from Herpesviruses. In particular TLR2 alllows NK-mediated recognition of envelope glycoproteins from HSV and CMV, while TLR9 can recognize viral CpG sequences shuttled by KIR3DL2 from the surface of NK cells to endosomes. APC-derived cytokines and reciprocal interactions with these immune cells (e.g., dendritic cells and macrophages) can further enhance NK cells effector function against Herpesviruses.

The main mechanisms by which NK cells can recognize and eliminate virus-infected cells involve the employ of (i) activating receptors for cellular ligands often overexpressed upon infection, (ii) activating receptors for virus-derived ligands, (iii) activating receptors, i.e., NKG2C and aKIRs, recognizing virus-modified HLA-I molecules, and (iv) CD16-mediated antibody-dependent cellular cytotoxicity (ADCC) (Hammer et al., 2018b). Almost all these mechanisms can be applied to NK cells in Herpesvirus control (Figures 1A–C).

The importance of certain activating receptors in Herpesvirus elimination has been indirectly revealed by the numerous proteins encoded by the different Herpesviruses aimed at limiting activating receptors function, in most cases by downregulating the respective cellular ligands on infected cells. In this context, the activating receptor NKG2D that recognizes stress-induced cellular ligands often overexpressed upon viral infection or tumor transformation (i.e., MIC-A, MIC-B, and ULBPs) (Lanier, 2015), is central in NK-mediated immune responses against virtually all Herpesviruses, namely HSV-1, VZV, CMV, HHV6, HHV7, KSHV, and EBV, all of which encode molecules downregulating NKG2D ligands (Wu et al., 2003; Thomas et al., 2008; Nachmani et al., 2009; Schneider and Hudson, 2011; Campbell et al., 2015; Schmiedel et al., 2016). Besides NKG2D, other non-HLA-I-specific activating receptors can play a role against several Herpesviruses suggesting a common strategy to eliminate these pathogens. In particular, the three NCRs (i.e., NKp46, NKp30, and NKp44) are involved in killing HSV-1-infected fibroblasts. The upregulation of cellular NCR ligands upon HSV-1 infection is resulted responsible for the increased susceptibility to NK-mediated cytotoxicity (Chisholm et al., 2007). Interestingly, NK-mediated killing was appreciable even before HLA-I downregulation had occurred, suggesting that, in NK-HSV-1 interactions, activating signals can overcome inhibitory receptors signaling (Chisholm et al., 2007). In this context, increased NCRs expression and function have been observed in NK cells differentiating *in vitro* from CD34^+^ precursors in the presence of HSV-infected myelomonocytes, further strengthening the relevance of the NCRs-NCR ligands axis against HSV (Costa et al., 2009).

The NCR NKp30 also participates in recognition and killing of CMV- and HHV6-infected cells. Its involvement is again testified by viral evasion mechanisms that downregulate B7-H6, a major NKp30 cellular ligand (Brandt et al., 2009), possibly expressed on infected cells (Schmiedel et al., 2016; Charpak-Amikam et al., 2017). In addition, NKp30 itself is the target of a CMV-encoded protein, pp65, that by binding to this NCR can induce its dissociation from the signaling molecule CD3ζ, thereby inhibiting NK-mediated killing of CMV-infected fibroblasts and dendritic cells (DCs) (Arnon et al., 2005). Along this line, a role for the NKp44-NKp44 ligand signaling pathway against KSHV is suggested by NKp44 ligand downregulation during lytic infection in KSHV-infected cells (Madrid and Ganem, 2012).

Similar to NKG2D and NCRs, the activating co-receptor DNAM1 recognizing PVR and Nectin-2 (CD112) (Bottino et al., 2003), plays a role against different Herpesviruses, i.e., CMV, EBV, and HSV-2 as demonstrated by different evasion strategies reducing DNAM-1 signaling (Tomasec et al., 2005; Prod’homme et al., 2010; Grauwet et al., 2014; Williams et al., 2015).

While NKG2D, DNAM-1, and NCRs serve against several Herpesviruses, other activating NK receptors are specifically involved in the recognition/elimination of cells infected only by a given Herpesvirus. An example is the co-receptor 2B4 (or CD244) which requires the adaptor protein SLAM-associated protein (SAP) to deliver activating signals upon engagement with its ligand CD48 (Nakajima et al., 1999; Bottino et al., 2000). 2B4 engagement is crucial to NK-mediated killing of EBV-infected B cells. Indeed, B cells that are CD48 high, represent a preferential target for this Herpesvirus (Chijioke et al., 2016). A role for 2B4 was actually revealed by the severe consequences of primary EBV infection in individuals suffering from X-linked lymphoproliferative disease (XLP-1), a congenital immunodeficiency in which SAP is absent or defective (Sayos et al., 1998), resulting in inhibitory signals from 2B4 impairing NK-mediated B-EBV elimination (Parolini et al., 2000). Interestingly, NK cells can respond efficiently to EBV-infected B cells in early lytic cycle and NK-mediated killing involves also NKG2D and DNAM-1 (Chijioke et al., 2013; Williams et al., 2015). However, EBV-infected B cells in latency or even in late lytic stages are resistant to NK attack, due to viral evasion mechanisms independent of NK cell function (Williams et al., 2015).

Finally, a role for the activating co-receptor NKp80 in the recognition of KSHV-infected cells was also proposed, based on the downregulation of its ligand AICL upon KSHV infection (Thomas et al., 2008).

Overall, in most instances, the activating receptors described above allow NK cells to eliminate infected cells by the recognition of cellular ligands expressed on target cells, while the engagement of activating receptors by virus-encoded ligands has not been demonstrated for Herpesviruses, at variance with influenza or vaccinia virus whose products hemagglutinin, and neuraminidase are directly recognized by NKp46 and NKp44 (Mandelboim et al., 2001; Ho et al., 2008). On the contrary, the HLA-I specific receptor NKG2C can recognize viral ligands although the mechanisms described so far are based on interactions with viral peptides bound to HLA-E molecules on CMV-infected cells. NKG2C is also involved in generating CMV-induced adaptive responses and will thus be discussed in more detail in the dedicated paragraph.

Another major mechanism employed by NK cells in controlling both primary viral infections, when adaptive immunity is already established, and secondary reactivations (either subclinical or clinical), relies on the activating receptor CD16 (FcγRIIIa), the low-affinity receptor for the immunoglobulin Fc fragment (Braud et al., 1998; Vivier et al., 2011). Upon CD16 engagement, NK cells can efficiently eliminate opsonized infected cells via ADCC. The relevance of this mechanism in providing defense against Herpesvirus is underlined by severe EBV and VZV infections associated to a dysfunctional mutated CD16 (de Vries et al., 1996; Grier et al., 2012). Furthermore, a polymorphism of the *CD16* gene resulting in the surface expression of a high affinity CD16 receptor (i.e., the CD16A-158V/V polymorphism) is associated to enhanced NK-mediated ADCC and confers protection from clinical HSV-1 reactivation (Moraru et al., 2012, 2015). Not unexpectedly, this highly effective anti-viral mechanism is targeted by multiple evasion strategies, as both HSV and CMV encode Fcγ-binding proteins that act as decoy receptors interfering with IgG binding to CD16 and thus attenuating ADCC (Johnson et al., 1988; Atalay et al., 2002; Corrales-Aguilar et al., 2014; Costa-Garcia et al., 2015). However, it has been recently described that a viral Fcγ-binding protein, gE, which is expressed on the cell surface by HSV-infected cells, can react with non-specific IgG thus generating a “Fc-bridge” that instead favors NK-mediated ADCC responses (Dai et al., 2017; Dai and Caligiuri, 2018).

NK cells can importantly contribute to early viral defense not only by exerting cytolytic activity against infected cells but also through their ability to sense pathogens via toll-like receptors (TLRs) (Sivori et al., 2004). NK cells express different functional TLRs among which TLR2, TLR3, and TLR9 seem to be primarily involved in the recognition of pathogen-associated molecular patterns (PAMPs) derived from Herpesviruses, such as double stranded viral nucleic acids or structural proteins (Adib-Conquy et al., 2014; Della Chiesa et al., 2014b). In particular, NK cells can directly recognize envelope glycoproteins from both CMV and HSV virions through TLR2 (Kim et al., 2012; Muntasell et al., 2013). Upon TLR2 engagement, NK cells become activated, and produce IFN-γ, further promoting anti-viral immune responses. Indeed, NK cells have been detected in herpetic lesions in close contact with CD4 T cells, thus possibly contributing to directly shaping adaptive responses (Kim et al., 2012). Interestingly, *TLR9* polymorphisms are associated with susceptibility to infection, with the T-1237C polymorphism that causes altered *TLR9* expression, being predictive of susceptibility to CMV infection (Carvalho et al., 2009). NK cells could thus play a role in TLR9-mediated defense to CMV, as they can efficiently respond to TLR9 agonists such as CpG-ODNs. Remarkably, these TLR9 ligands can be bound at the cell surface by KIR3DL2, a member of the KIR family, and then shuttled by receptor internalization to endosomes where TLR9 is localized (Sivori et al., 2010).

Thus, in a scenario where NK cells are recruited to viral infection sites, their effector function (e.g., cytotoxicity, IFN-γ, and chemokine production) can be enhanced by combined exposure to microbial products and cytokines available in the inflammatory milieu, such as IL-12 or IL-18. In this context, TLRs- and/or cytokine-activated NK cells can reciprocally interact with other immune cells responding to the same PAMPs via TLRs, such as DCs or macrophages (Figure 1D). This cross-talk can occur in the early phases of anti-viral responses (Andrews et al., 2005; Vogel et al., 2014) and can also contribute to DC editing and/or promote DC maturation (Della Chiesa et al., 2005, 2014b; Ferlazzo and Morandi, 2014), thus possibly amplifying and regulating adaptive responses to Herpesviruses.

It should be noted however, that TLR-mediated sensing of viral PAMPs by NK cells has not been definitively settled yet, similar to the contribution of TLRs on DC and macrophages to the response to NK cells. A more extensive review work and additional original work will be needed to appropriately address this issue.

## “Adaptive” Nk-Cell Responses to Cmv

The conventional view of NK cells as short-lived innate lymphocytes, unable to retain any kind of memory has been considerably challenged in the last years, based on several studies demonstrating that NK cells are capable of adapting to viruses and keep memory of past infections (Sun and Lanier, 2009; Sun et al., 2011, 2014; Della Chiesa et al., 2015, 2016). Interestingly, the first evidence that NK cells can develop memory responses to pathogens was against the Herpesvirus CMV, initially in mice (Hadinoto et al., 2009) and later on in humans (Della Chiesa et al., 2012; Foley et al., 2012b; Muccio et al., 2016).

In CMV-seropositive individuals a striking expansion of NK cells expressing the HLA-E-specific activating receptor CD94/NKG2C was observed 15 years ago (Guma et al., 2004). Further studies on NK cells developing in hematopoietic stem cell transplation (HSCT) recipients showed that indeed CMV is a powerful driver of NK cell differentiation favoring the expansion of KIR^+^NKG2A^–^LILRB1^+^ mature NK cells expressing the marker of terminal differentiation CD57 (Della Chiesa et al., 2012; Foley et al., 2012a, b; Locatelli et al., 2018).

In the HSCT setting the CMV-induced reconfiguration also revealed features typical of adaptive immunity, i.e., virus-induced clonal expansions and long-term persistence that led to the concept of “adaptive” or “memory” NK cells (Sun et al., 2011; Della Chiesa et al., 2016; Rolle and Brodin, 2016). This peculiar CMV-driven NK cell subset is characterized by epigenetic modifications, altered expression of signaling molecules and transcription factors that modulate their phenotype and function (Luetke-Eversloh et al., 2014; Lee et al., 2015; Schlums et al., 2015). The generation of this population likely involves interactions between NKG2C and its ligand HLA-E that usually binds peptides derived from HLA-I leader sequences. However, in CMV-infected cells, HLA-I molecules are downregulated by viral evasion mechanisms, while HLA-E can be stabilized and upregulated by peptides derived from the viral-encoded protein UL40 leader sequence, thus stimulating NKG2C^+^ NK cells and favoring adaptive NK cells expansion (Guma et al., 2006; Rolle et al., 2014). Interestingly, recent studies demonstrated that NKG2C^+^ NK cells can distinguish subtle differences between peptides bound to HLA-E molecules, showing stronger responses to a particular peptide derived from rare variants of CMV-encoded UL40, precisely mimicking the peptide derived from HLA-G leader sequence (Hammer et al., 2018a; Rolle et al., 2018). This peptide-specificity and the avidity selection of NK cells during CMV infection recently reported in mice (Adams et al., 2019), further support the concept that CMV recognition by NK cells can elicit responses akin to T cell-adaptive responses.

In addition to NKG2C-HLA-E interactions, CD2-costimulation, and different cytokines such as IL-12, IL-18, and IL-15 are involved in adaptive NK cells generation and proliferation (Hammer et al., 2018a; Rolle et al., 2018).

Upon CMV-induced reconfiguration, NK cells display specialized effector function, showing in particular enhanced ADCC abilities. This increased response to Ab-coated targets has been associated to the downregulated expression of the signaling protein FcεRγ which represents a common feature in CMV-adapted NK cells (Lee et al., 2015; Schlums et al., 2015; Muntasell et al., 2016; Muccio et al., 2018). Although the generation of this subset seems to be promoted exclusively by CMV, its increased ability to eliminate Ab-coated infected cells through enhanced ADCC could keep under control infections and reactivations caused by other viruses, as suggested by studies reporting efficient ADCC-mediated killing of opsonized EBV- and HSV-infected targets by adaptive NKG2C^+^ NK cells (Costa-Garcia et al., 2015; Moraru et al., 2015).

Interestingly, adaptive NKG2C^+^ NK cells are also capable of presenting CMV antigens through HLA-DR to autologous memory CD4 T cells (Costa-Garcia et al., 2019), regulating T-cell mediated adaptive responses to CMV and possibly contributing to control viral reactivations.

Besides the central role played by NKG2C, aKIRs are also involved in CMV recognition and generation of adaptive responses (Beziat et al., 2013; Della Chiesa et al., 2015). Indeed, CMV infection can promote the expansion of mature NK cells expressing aKIRs in patients receiving Umbilical Cord Blood transplants from NKG2C^–/–^ donors, thus lacking NKG2C expression (Della Chiesa et al., 2014a). The involvement of aKIRs is in line with observations in mice where NK cells expressing the activating receptor Ly49H, homolog of aKIR, expand in response to MCMV infection and confer long-term protection to secondary challenges through the recognition of the viral-encoded ligand m157 (Arase et al., 2002; Hadinoto et al., 2009). Moreover, in humans, a reduced risk of CMV reactivation was associated to the presence of aKIRs in both hematological and solid organ transplant patients supporting their role in anti-viral defense (Stern et al., 2008; Zaia et al., 2009; Mancusi et al., 2015). The exact mechanisms underlying the recognition of infected cells by aKIRs has not been precisely elucidated, however a role for KIR2DS1 in the recognition of its ligand HLA-C2, modified by CMV in infected fibroblasts, has been recently reported (van der Ploeg et al., 2017). Interestingly, KIR2DS1 tetramers were also described to efficiently interact with EBV-infected B cells expressing HLA-C2 (Stewart et al., 2005; Figure 1C).

Notably, in individuals lacking both NKG2C and aKIRs, CMV infection can still favor NK cell reconfiguration indicating that additional unknown mechanisms are responsible for CMV recognition and adaptive NK cell differentiation (Muntasell et al., 2016).

While in mice it has been reported that NK cells can maintain memory of prior encounters with HSV-2 and protect from reactivations (Abdul-Careem et al., 2012), in humans few reports suggest that Herpesviruses other than CMV can induce the generation of specific NK cell subsets with memory properties. Upon EBV infection an expansion of CD56^bright^NKG2A^+^CD62L^–^ NK cells was observed in tonsils (Lunemann et al., 2013), whereas CD56^dim^NKG2A^+^KIR^–^ NK cells accumulated in peripheral blood during infectious mononucleosis and were involved in lytic EBV-infected B cells elimination (Azzi et al., 2014). However, at variance with CMV-induced expansions, EBV-induced NK cells were not bearing a specific activating receptor and evidences for their epigenetic reprograming has not been provided (Chijioke et al., 2016).

Further studies are necessary to investigate the impact of NK-Herpesvirus interactions in inducing adaptive NK cell subsets outside the CMV context. The possibility to generate virus-specific NK cell populations could help in designing novel vaccine protocols against Herpesviruses, considering that only anti-VZV vaccines have been successfully developed (Arnold and Messaoudi, 2017). However, in the generation of novel vaccines, it should be considered that prolonged exposure to both VZV and HSV-1 can directly impair NK-cell effector function, through still unknown mechanisms, as recently described (Campbell et al., 2019).

## Clinical and Biological Perspective and Concluding Remarks

As mentioned above, major defects in NK cell function in respect to human Herpesviruses have been described and become overwhelmingly manifest during primary infections that may be lethal upon first host-virus encounter [e.g., SAP defects, NK cell deficiencies (Sayos et al., 1998; Orange, 2002; Etzioni et al., 2005; Notarangelo and Mazzolari, 2006; Mace and Orange, 2019)]. These cases represent a very limited part of Herpesvirus-induced clinical syndromes, since most primary infections are controlled by the immune system often as asymptomatic infections and latency ensues in the vast majority of patients without further clinical reactivations in >70% of infected subjects in the absence of secondary immunodeficiencies (e.g., HIV infection, transplantation, immunosuppression) (Clark and Griffiths, 2003; Ljungman et al., 2011; Locatelli et al., 2016). For this reason, most NK cell evasion mechanisms are less relevant during this acute phase of primary infection. Herpesvirus latency (e.g., HSV/VZV in neuronal ganglia, EBV in B cells and epithelial cells or CMV in organ and BM macrophages) has been long considered a period of antigenic eclipse to the immune system, while reactivation with clinical symptoms (e.g., recurrent HSV, Zoster or shingles, transformation by EBV or KSHV) represent a possible failure of the immune system to control viral latency. Most virus-induced strategies to evade NK cell (and/or T cell) control may be active during these “clinical escape” or reactivation phases. This perspective, however, needs to be carefully reevaluated in view of the overwhelming evidence showing that exit from latency or virus reactivation routinely occurs for all Herpesviruses in infected hosts at subclinical levels (Ling et al., 2003; Hadinoto et al., 2009; Schiffer et al., 2009; Tronstein et al., 2011). Thus, clinically latent Herpesvirus infection actually has a continuous component of persistent immune stimulation due to virus replication in part of the infected cells pool. In this context, virus evasion mechanisms are likely to occur continuously, and are quantitatively more frequent and relevant than during primary infection. Indeed, the magnitude of the specific T cell response during CMV clinical latency is surprisingly high, with 10–20% of CD4 and CD8 CMV-specific circulating T cells, and 5–15% of NKG2C^+^ memory-like NK cells during clinical latency (Guma et al., 2004; Sylwester et al., 2005). For example, during latent EBV infection 5–10% of peripheral CD8 T cells are specific for latent or lytic epitopes (Tan et al., 1999; Hislop et al., 2002) and 20% of tonsil lymphocytes are EBV-specific (Hislop et al., 2005).

In view of these considerations, and of the participation of persistent Herpesvirus infection to the modulation of autoimmune, allergic, atopic and atherosclerotic events, Herpesviruses and the host may be regarded from an evolutionary-ecologic perspective as co-evolved symbionts with an evolutionary relationship (Virgin et al., 2009; Roossinck, 2011). It will be critical for future scientific focus to more precisely dissect which NK cell evasion mechanisms are functional to maintain this symbiontic equilibrium, from those that actually determine more severe, clinically relevant reactivations, particularly in immunosuppressed patients or in those with virus-induced tumor (e.g., NHL and KS).

## Author Contributions

All authors listed have made a substantial, direct and intellectual contribution to the work, and approved it for publication.

## Conflict of Interest

The authors declare that the research was conducted in the absence of any commercial or financial relationships that could be construed as a potential conflict of interest.
